# Characteristics of Composite Deprivation Indices Used in Public Health: A Scoping Review Protocol

**DOI:** 10.3390/ijerph191710565

**Published:** 2022-08-24

**Authors:** Anastasia Zelenina, Svetlana Shalnova, Sergey Maksimov, Oksana Drapkina

**Affiliations:** National Medical Research Center for Preventive Medicine of the Ministry of Healthcare of the Russian Federation, Petroverigskiy per. 10, 101990 Moscow, Russia

**Keywords:** deprivation index, residence characteristics, taxonomy, scoping review, protocol, health inequality

## Abstract

**Introduction:** A deprivation index has become a more popular tool to rank levels of deprivation within different geographic areas. It is extensively used for monitoring health inequalities, evaluating health care services, developing and modifying health policies and programs, and allocating health resources equitably. Our objectives are (1) to explore the relevant literature to describe features of composite deprivation measures; (2) create a list and classification of original deprivation indices. We will develop the classification of indices to systematize knowledge and improve the functional utility of the information. **Methods:** Any original deprivation index mentioned in peer-reviewed or grey literature documents will be eligible for inclusion if it assesses deprivation at a population level and used in relation to health. The study area will be limited to the geographic areas of North America, Europe, Australia, and New Zealand. Tables and a narrative summary will be used to describe features of deprivation indices. Diagrammatic form will be used to create the classification of deprivation indices. **Discussion:** Practically, the results of this study could facilitate finding a common language among researchers and specialists who create and use deprivation indices, thus helping the development and implementation of appropriate deprivation indices for different countries.

## 1. Introduction

In 1979, Townsend defined “deprivation” as the concept of unmet needs due to an actual or perceived lack of resources required to maintain the quality of life (e.g., diet, activities, material benefits) to which various socioeconomic groups or individuals within those groups have grown accustomed, or are considered to be the accepted norm within the group [1]. A deprivation index is an instrument to measure and categorize levels of deprivation across different geographies to evaluate the influence of the social and physical environment on health behaviors and outcomes.

Although researchers also utilize individual variables reflecting material, social [2,3] and environmental [4] deprivation, the development and use of composite indices for capturing diverse multidimensional deprivations are becoming increasingly popular. Deprivation indices have both benefits and limitations. On the one hand, they are easily comprehensible, multipurpose tools and hence, they are widely used in medical practice, research, and policymaking.

A number of epidemiological studies have been carried out around the world that examine the effect of deprivation on a wide range of health measures and disparities. Overwhelming evidence shows major negative impacts of deprivation on human health, for instance, type 2 diabetes and obesity in Germany [5] and South Korea [6], cardiovascular diseases and risky health behaviors in the United States [7], England [8], and China [9], mortality rate among adults in Spain [10] and France [11], premature mortality in Cyprus [12], Canada [13], mental disorders in the United States [14].

The deprivation indices are commonly used in public health research to quantify social inequalities in health and environmental health inequality, or to assess the effect of some characteristics of areas (economic, social, and environmental) on health behaviors and outcomes separately. In addition, local health authorities use these indices to help identify areas where the need for medical care and the demand for medical services are expected to be the highest and then target health policies and programs to the areas in order to achieve the following goals: to improve health outcomes, to allocate financial and technical resources appropriately and to make health services broadly accessible.

However, despite their considerable advantages, deprivation indices are unique and country specific. Therefore, in creating a deprivation index, it is important to take into account differences in the social, cultural and economic characteristics, such as income, employment, social supports, community safety and other relevant factors of different countries. In addition, the development of an index depends on technical progress and socio-economic changes in any country. For example, ownership of a color TV is not an indicator of deprivation for high-income countries, as the majority of the population living there has this device, but the variable may be essential to calculate levels of deprivation in low-income countries.

Some researchers have imported slightly modified publicly available indices, such as the Townsend index [15], and the Carstairs index [16], which were developed for other countries, rather than design specific instruments per se, but these do not always reflect the concept of deprivation in a particular country. The use of contradictory and less reliable deprivation measures may lead to a significant underestimation of the association of deprivation with health outcomes.

In the current review, we will examine how, in different countries, deprivation indices were created. This is significant because indices have been used in public health for more than three decades. The first deprivation index was created in the UK in the late 1980s. A stock of knowledge and information about deprivation indices have been accumulated over this period. If in the past they were only used to measure material and/or social deprivation (e.g., income gap, high illiteracy rate, low level of education, poor quality of housing, single-parent families), now they are created from different domains, which also include indicators relating to environmental deprivation (e.g., air pollution, noise levels, unsafe neighborhood streets and parks, and access to environmental resources such as green or blue spaces). Furthermore, deprivation indices are included in other composite measures, such as the Health Opportunity Index (HOI) [17] that cover all aspects of human activity (social, economic, educational, demographic, and environmental factors) which affect human health and well-being.

However, the scientific community still cannot reach a consensus on selection of appropriate data sources (census, survey, and administrative data), individual deprivation indicators and geographic areas for creating an index. In this regard, we will create a taxonomy of indices to systematize knowledge and improve the functional utility of the information. Moreover, the classification will provide an organized framework for planning and following through with the process of developing new deprivation measures. Therefore, the findings of the review will serve as a valuable resource material for future studies, programs, plans and projects.

In the light of the above, the purpose of this scoping review will be to investigate the nature and characteristics of deprivation indices. The following specific objectives have been pursued: (1) to review the relevant literature to describe features of composite deprivation measures (data sources, weighting methods, etc.); (2) create a list and taxonomy of deprivation indices.

A preliminary search of PubMed found a recently published scoping review from 2018 by Ichihara et al. on area deprivation measures used in Brazil [18]. Ichihara et al. included 30 articles. The main goals of this scoping review were to inform the development of a small-area deprivation index for Brazil and review the literature to describe currently used area-based measures of socioeconomic inequalities in Brazil. In contrast to Ichihara et al., this review will identify and evaluate the literature on original deprivation indices that exist globally in order to create a taxonomy. A preliminary search of PROSPERO and the JBI Evidence Synthesis was conducted and no current or underway scoping reviews or systematic reviews on the topic were identified.

## 2. Methods

The proposed scoping review will be conducted in accordance with the JBI methodology for scoping reviews [19]. We have not registered this review with PROSPERO, because scoping reviews are not registered with PROSPERO. The PRISMA-P checklist is included in the Supplementary Materials.

### 2.1. Review Question(s)

1. Which original deprivation indices (used in public health) exist around the world?
2. What area levels (country/area of country) are the indices operationalized at?
3. What years and data sources are used to create the indices?
4. Which deprivation indicators/domains make up the indices?
5. Which types of deprivation (social, material, health, and environment) are considered in the indices?
6. Which weighting methods are used to create the indices?
7. Are updated versions of deprivation indices available?
8. Are separate websites and/or supporting documentation about the indices available?

### 2.2. Inclusion/Exclusion Criteria

Study selection will be performed based on the inclusion/exclusion criteria. The eligible article should indicate the following: (1) the territory for which an index was created, (2) a list of all the indicators that form the index, (3) the data source from which these indicators were extracted, (4) the weighted method used to assess the importance of the indicators in the index. In addition to information about creating an index, (5) the article should present evaluation of the predictive validity of the index (the relationship between deprivation and health) [20] or evidence that researchers plan to do so in the near future. The review will include only the very first eligible article.

The concept of this review is to create a taxonomy of deprivation indices. Therefore, this review will consider studies where researchers created an original deprivation index to examine the effect of deprivation on health. An original deprivation index is defined as “an original deprivation index that includes a combination of deprivation indicators that is unique and not repeated in other indices”.

This review will consider the deprivation indices developed for both a whole country (e.g., national deprivation indices in Canada) and specific areas of a country (e.g., Palmetto Small-Area Deprivation Index (Palmetto SADI)). The study area will be limited to the geographic areas of North America, Europe, Australia, and New Zealand.

Observational studies (ecological, cross-sectional, case-control, and cohort) will be eligible for inclusion.

Questionnaire-based individual-level deprivation indices will be excluded (e.g., a deprivation in primary care questionnaire (DiPCare-Q index), a New Zealand index of socioeconomic deprivation for individuals). Adapted (e.g., indices based on the Townsend Index) and updated versions of deprivation indices will be excluded. We define an adapted index as “an index (containing a certain (unique) set of deprivation indicators) that is applied outside the country for which it was created”. An updated index is defined as “an updated index that is already available, but undergoes transformation over time (elimination or addition of deprivation indicators, taking into account social and economic changes in the country)”.

Index creation guidelines, systematic, scoping and other reviews will be excluded. Experimental studies (i.e., crossover studies, health education programs, randomized control trials, laboratory science studies), conference proceedings, editorials, commentaries, and duplicate publications will be excluded.

### 2.3. Search Strategy

The search strategy will aim to locate only published studies, without publication date limit. Papers published in any language will be included to avoid language bias. On 10 December 2020, an initial limited search of MEDLINE (PubMed) was undertaken to identify articles on the topic. A total of 1040 results were obtained. Bibliographic databases will be searched to find papers that include “deprivation index”, “deprivation indices”, or “deprivation indexes” in the title and/or abstract field. There will be no restriction by publication year. The reference lists of papers included in the review will be screened for additional papers.

### 2.4. Information Sources

The databases to be searched include MEDLINE (PubMed). Sources of gray literature to be searched include Google Scholar. For each included deprivation index, we will use a Google search to determine if there is a separate website and/or supporting documentation about the index and its methodology to establish whether it was used outside research projects. In the same way, we will search for updated versions of indices. In our opinion, the parameters (such as availability of separate website, supporting documentation, and updated version) clearly demonstrate the simplicity of use, relevance, and accessibility of the index. If the information is only available via the included study, then the data will be extracted from there. The information source will be recorded as part of the data extraction.

Optionally, the search of relevant studies that are already known to the authors will be included in the scoping review.

### 2.5. Study Selection

Following the search, all identified records will be collated and uploaded into EndNote X9 (Clarivate Analytics, Philadelphia, PA, USA) and duplicates removed. Following a pilot test, titles and abstracts will then be screened by two independent reviewers (A.Z. and S.M.) for assessment against the inclusion criteria for the review. Potentially relevant papers will be retrieved in full and their citation details imported into the JBI System for the Unified Management, Assessment and Review of Information (JBI SUMARI; JBI, Adelaide, Australia) [21]. The full text of selected citations will be assessed in detail against the inclusion/exclusion criteria by two independent reviewers. Reasons for exclusion of full-text papers that do not meet the inclusion criteria will be recorded and reported in the scoping review. To select only the original indices and exclude the adapted and updated indices, at the full-text screening stage the earliest published papers will be first read. Any disagreements that arise between the reviewers at each stage of the selection process will be resolved through discussion or with a third reviewer (S.S.). The results of the search will be reported in full in the final scoping review and presented in a Preferred Reporting Items for Systematic Reviews and Meta-analyses for Scoping Reviews (PRISMA-ScR) flow diagram [22].

### 2.6. Data Extraction

Data will be extracted from papers included in the scoping review by two independent reviewers (A.Z. and S.M.) using a data extraction form developed in Microsoft Excel by A.Z. (see Appendix A). The data extracted will include specific details about the population, concept, context, methods, and key findings relevant to the review questions. The following information will be extracted for each included study: bibliographic details, years of study, spatial scale (characterizes the scale of the territory where the index is used), data source, weighting method, and updated versions of deprivation indices. The draft data extraction form will be modified and revised as necessary during the process of extracting data from each included paper. Modifications will be detailed in the full scoping review. Any disagreements that arise between the reviewers will be resolved through discussion or with a third reviewer (S.S.). Authors of papers will be contacted to request missing or additional data, where required.

### 2.7. Data Analysis and Presentation

The extracted data will be presented in diagrammatic and tabular form in a manner that reflects the objective and scope of this scoping review. A narrative summary will accompany the tabular and/or charted results and will describe how the results relate to the review’s objective and questions. Tables and figures will be used where appropriate.

Data on the deprivation indices will be analysed to create a list and taxonomy of indices that will be available to researchers, practitioners, and policymakers. This will include quantitative analysis of the indicators and domains that make up indices, for instance, the percentage of indices measuring particular features (material, social, environmental, health deprivation). The analysis will also assess the proportion of indices operating in particular geographic scales and the proportion of indices developed using different variable/domain weighting methods.

## 3. Discussion

The ambiguous nature of indices makes it difficult to fully recognize their potential in both scientific studies and practical applications. Accordingly, it is important to explore the development of indices in greater depth. Based on a description of features of deprivation measures as well as a methodology and interpretation of indices, we will create their taxonomy. The aim of creating the taxonomy is to systematize information related to the methodology for constructing and using deprivation indices in public health research and practice. Practically, the results of this study could facilitate finding a common language among researchers and specialists who develop and use deprivation indices, thus helping the development and implementation of appropriate deprivation indices for different countries.

## 4. Conclusions

We hope that the data from the review will stimulate the use of a competent approach and will help researchers and public health specialists in resolving conflicts or inconsistencies that arise during the construction and use of indices.

## Data Availability

No new data were created or analyzed in this study. Data sharing is not applicable to this article.

## Supplementary Materials

The following supporting information can be downloaded at: https://www.mdpi.com/article/10.3390/ijerph191710565/s1, Table S1: PRISMA-P (Preferred Reporting Items for Systematic review and Meta-Analysis Protocols) 2015 Checklist: Characteristics of composite deprivation indices used in public health: a scoping review protocol [23].

## Appendix A

**Table A1:** Main characteristics of deprivation indices.

Name of Index/Reference (Year of Publication)	Country (Area of Country)	Data Source (Geographical Unit)	Weighting Method	Updates	Website and/or Supporting Documentation
					

**Table A2:** Indicators of deprivation measures.

Name of Index/Reference (Year of Publication)	Variables/Subdimensions	Domains/Dimensions	Type of Deprivation
			
